# Exclusive Enteral Nutrition Beneficially Modulates Gut Microbiome in a Preclinical Model of Crohn’s-like Colitis

**DOI:** 10.3390/nu16030363

**Published:** 2024-01-26

**Authors:** Ramasatyaveni Geesala, Neeraja Recharla, Ke Zhang, John C. Johnson, George Golovko, Kamil Khanipov, Douglas L. Brining, Xuan-Zheng Shi

**Affiliations:** 1 Department of Internal Medicine, The University of Texas Medical Branch at Galveston, Galveston, TX 77555, USA; rageesal@utmb.edu (R.G.); nerechar@utmb.edu (N.R.); kezhang@utmb.edu (K.Z.); johcjohn@utmb.edu (J.C.J.); 2 Department of Pharmacology & Toxicology, The University of Texas Medical Branch at Galveston, Galveston, TX 77555, USA; gegolovk@utmb.edu (G.G.); kakhanip@utmb.edu (K.K.); 3 Department of Microbiology & Immunology, The University of Texas Medical Branch at Galveston, Galveston, TX 77555, USA; dlbrinin@utmb.edu

**Keywords:** exclusive enteral nutrition, liquid diet, Crohn’s disease, microbiota, colitis, dysbiosis

## Abstract

Exclusive enteral nutrition (EEN) is an established dietary treatment for Crohn’s disease (CD) by alleviating inflammation and inducing remission. However, the mechanisms of action of EEN are incompletely understood. As CD is associated with gut microbiome dysbiosis, we investigated the effect of EEN on the microbiome in a rat model of CD-like colitis. The rat model of CD-like colitis was established by an intracolonic instillation of TNBS at 65 mg/kg in 250 µL of 40% ethanol. Sham control rats were instilled with saline. Rats were fed ad libitum with either regular pellet food or EEN treatment with a clear liquid diet (Ensure). Rats were euthanized at 7 days. Fecal pellets were collected from the distal colon for 16S rRNA sequencing analysis of gut microbiota. In addition, colon tissues were taken for histological and molecular analyses in all the groups of rats. EEN administration to TNBS-induced CD rats significantly improved the body weight change, inflammation scores, and disease activity index. The mRNA expression of IL-17A and interferon-γ was significantly increased in the colonic tissue in TNBS rats when fed with regular food. However, EEN treatment significantly attenuated the increase in IL-17A and interferon-γ in TNBS rats. Our 16S rRNA sequencing analysis found that gut microbiota diversity and compositions were significantly altered in TNBS rats, compared to controls. However, EEN treatment improved alpha diversity and increased certain beneficial bacteria such as *Lactobacillus* and *Dubosiella* and decreased bacteria such as *Bacteroides* and *Enterorhabdus* in CD-like rats, compared to CD-like rats with the regular pellet diet. In conclusion, EEN treatment increases the diversity of gut microbiota and the composition of certain beneficial bacteria. These effects may contribute to the reduced inflammation by EEN in the rat model of CD-like colitis.

## 1. Introduction

Inflammatory bowel disease (IBD), including ulcerative colitis (UC) and Crohn’s disease (CD), is chronic inflammation in the intestine with symptoms such as diarrhea and abdominal pain [1]. The etiology of IBD is multifactorial and largely unknown. However, an increasing trend in the incidence and prevalence of IBD depicts genetics, gut microbes, and dietary and environmental factors to be the major contributing factors. Among these, dietary factors in early life events such as maternal diet, antibiotics, and formula feeding play a significant role in the development of gut microbiota. And gut microbiota dysbiosis is found to be closely associated with CD and UC [2]. Several studies from the past two decades have revealed that the microbiome in the gastrointestinal tract is crucial for maintaining gut homeostasis. Physiologically, gut microbial interactions with the host help with gut epithelial barrier maintenance, vitamin production, nutrient metabolism, and immune response mechanisms [3]. However, altered host–microbiome interactions in the gut during CD may lead to dysregulated immune responses [2]. Although there is an advancement in therapeutic strategies in IBD, CD still presents a constant challenge due to its associated co-morbidities. The conventional treatments for CD are mostly based on targeting immunomodulation and inflammatory cytokines. These therapeutic strategies have shortcomings such as adverse effects and non-adherence. Therefore, there is a dire need to develop dietary treatments that can positively impact the gut microbiome to achieve therapeutic benefits in CD.

Several dietary strategies have been tested in CD patients. These include partial enteral nutrition (PEN); a specific carbohydrate diet (SCD); a Crohn’s disease exclusion diet (CDED); low fermentable oligosaccharides, disaccharides, monosaccharides, and polyols (FODMAP); and exclusive enteral nutrition (EEN) [4]. Among these, EEN is the only established treatment for CD, and it has been recommended by the European Society of Pediatric Gastroenterology, Hepatology and Nutrition (ESPGHAN) and the European Crohn’s and Colitis Organization (ECCO) [5] for clinicians to use as a first line of therapy to CD patients, especially pediatric patients. As the name presents, EEN represents a complete liquid diet either through an oral or intragastric route for a period of 6-8 weeks with a specialized nutritional component. EEN has shown strong evidence of remission in different stages of pediatric CD patients. Also, EEN has shown advantages over the shortcomings of long-term steroid use and the lower adherence of inflammatory modulators in CD patients [6]. Moreover, studies have reported that EEN not only induces remission in CD but also reduces inflammation [7,8,9].

Although EEN has been shown to be a promising dietary treatment, its mechanism of action is still ambiguous. Various theories have been deciphered to understand how EEN exerts its beneficial effects, which include modulation of the inflammatory cellular milieu [10] and maintenance of the intestinal barrier integrity [11]. EEN administration was shown to significantly reduce inflammatory gene expression and improve the balance of pro- and anti-inflammatory cytokines [9,12]. An in vitro study revealed that EEN reduced the expression of inflammatory cytokine IL-8 through regulating the NF-κB signaling pathway [13]. Other reports demonstrated an improved epithelial permeability [14]. Our recent studies have revealed that EEN may exert its beneficial effects by eliminating mechanical stress and attenuating mechanical stress-induced pro-inflammatory mediators in CD [15]. However, the effect of EEN on the gut microbiome has not been well addressed in these reports. Earlier studies focusing on gut microbiota changes after EEN treatment revealed that EEN might have widely changed the gut microbial compositions of CD patients [16]. Most of those reports found that EEN reduced the diversity of microbial organisms in the gut [17,18]. Those are human studies in which patients may have various extents of inflammation in different parts of the gut, and the exact diets or enteral nutrition may not be well controlled.

To investigate how EEN modulates the gut microbiome as well as gut inflammation, we used a standardized rodent model of Crohn’s-like colitis. Crohn’s-like colitis was induced by an intracolonic instillation of TNBS 65 mg/kg in 250 µL of 40% ethanol [12,19]. As reported previously [12,15], this treatment induces transmural and stenotic inflammation mimicking Crohn’s disease in the colon. Sham control rats were treated with an intracolonic instillation of 250 µL of saline. To study the effects of EEN treatment with a liquid diet, the sham control and CD rats were fed with either regular pellet food or exclusively in EEN with a liquid diet (Ensure) after the induction of colitis. We found that gut microbiota was significantly altered in CD rats compared to sham control rats. EEN treatment beneficially modulated the gut microbiome by increasing the abundance of some beneficial bacteria including *Lactobacillus*, and *Dubosiella*, and decreased detrimental bacteria such as *Escherichia–Shigella*. Our study signifies that EEN-mediated gut microbial alteration can be one of the underlying mechanisms of action of EEN in CD remission.

## 2. Methods

### 2.1. Animals

Male 8- to 10-week-old Sprague Dawley rats were utilized to conduct this study. All the rats were housed and bred at the animal facility of the University of Texas Medical Branch (temperature, 23 °C; humidity 55% ± 10; 12 h light/dark cycle) with free access to food and water throughout this study. All the experiments were performed with approval from the institutional animal care use committee and according to the committee guidelines (#0907051D).

### 2.2. Induction of Crohn’s-like Colitis and EEN Treatment

Crohn’s-like colitis was induced in 8- to 10-week-old Sprague Dawley rats by intracolonic instillation of 250 µL of TNBS (65 mg/kg in 40% ethanol). Sham control rats were treated with saline instillation. The instillation procedure was performed by utilizing a catheter inserted 7 cm inside of the anus, as previously described [12,15]. Before the TNBS or saline instillation, rats were fasted and treated with bowel cleanser for 24 h. The saline- or TNBS-treated rats were fed ad libitum either with regular pellet food (Picolab Rodent Diet 5053) or EEN liquid diet (Ensure Clear; Abbott, Abbott Park, IL, USA) for 7 days. The four groups of rats (EEN, sham, TNBS, TNBS + EEN) were euthanized on day 7. Full-thickness tissues from the distal colon (7~8 cm from the anus) in all the groups of rats were collected for molecular and functional analysis [20,21]. Fecal pellets were collected from the distal colon for microbiome analysis [20]. Body weight changes, inflammation score, and the disease activity index (DAI) (on a 6.0 scale) were determined as described earlier [15].

### 2.3. Colonic Tissue Preparations and Quantitative Real-Time Polymerase Chain Reaction

Total RNA was isolated from the colonic tissues from all the four groups of rats using RNeasy Mini Kit according to the manufacturer’s protocol (Qiagen, Valencia, CA, USA) [21], and reverse transcribed to cDNA with Bio-Rad iScript reverse transcriptase (Bio-Rad). Real-time quantitative RT-PCR was performed using the Bio-Rad CFX9000 Thermocycler (Hercules, CA, USA), as described previously [15,21]. TaqMan probes for interleukin-17 (IL-17) (Rn01757168_m1) and interferon-gamma (IFN-γ) (Rn01649192_m1) were purchased from Thermo Fisher Scientific (Waltham, MA, USA). The fold-change relative to control was calculated with the comparative CT (ΔΔCT) method with endogenous reference 18S rRNA (Part no. 4352930E, Applied Biosystems, Waltham, MA, USA) as the normalizer.

### 2.4. Microbiome Analysis

#### 2.4.1. Fecal DNA Extraction

Upon euthanization, fecal contents were collected from the distal colon and snap-frozen in liquid nitrogen. Fecal bacterial DNA was extracted using a MoBio PowerFecal kit (MoBio, Carlsbad, CA, USA) according to the manufacturer’s guidelines [20,22]. Isolated DNA concentrations were measured by spectrophotometry (BioTek Synergy H4 Hybrid Reader) and stored at −80 °C in a freezer.

#### 2.4.2. 16S rRNA Sequencing

Fecal DNA isolates from each sample were utilized to generate their sequencing libraries using the Quick-16S Plus NGS Library Prep Kit (D6420, Zymo Research, Irvine, CA, USA), which targets the universal 16S rRNA V3-V4 hypervariable regions (341f (CCTACGGGDGGCWGCAG, CCTAYGGGGYGCWGCAG, 17 bp and 806r (GACTACNVGGGTMTCTAATCC, 24 bp). Sequencing was performed with an Illumina MiSeq instrument using a MiSeq 600-cycle paired-end reagent kit v3 (MS-102-3003, Illumina, San Diego, CA, USA). Trimming of raw sequencing reads to 300 bases was performed to exclude reads with unknown nucleotides or sequencing adapters [22]. To identify the known bacteria, the sequences were analyzed using CLC Genomics Workbench 23.0.3 Microbial Genomics Module (CLC MGM). Sequenced reads were quality-controlled to trim reads with 2 or more unknown nucleotides or sequencing adapters. The 300-base paired-end reads were merged based on the overlap. Reference-based OTU picking was performed using the SILVA SSU v1138.1 99% sequence identity [23]. Sequences present in more than one copy but not clustered to the database were placed into de novo OTUs (99% similarity) and aligned against the database with an 80% similarity threshold to assign the closest taxonomical name where possible. OTUs with an abundance of less than 2 were taken out from the analysis. Abundance profiling was performed using MicrobiomeAnalyst 2.0 [24] at various taxonomic levels with actual and total sum scale abundance.

Alpha diversity was measured using the observed number of features, Chao1, Shannon diversity index at genus level to describe the identified metagenomic richness and diversity. Beta diversity was measured using the Bray–Curtis dissimilarity at the genus level. Bar plots were generated using the median ratio (log 2) with hierarchical clustering on the genera by their Euclidian distance using Ward’s method [25]. The comparisons were performed at both genus and phylum levels where statistical significance was calculated using the Mann–Whitney test, not assuming a specific data distribution.

Mean relative abundance was used to calculate the total relative abundance percentage among the samples at genus and phylum levels. The Wald test was used to determine significance between groups. The CLC MGM analysis used the trimmed mean of M-values instead of total sum scaling and a Bonferroni-corrected value of p cutoff of 0.05.

Linear discriminant analysis (LDA) effect size (LEfSe) was performed to identify the genera differentially represented in all the four groups of rats. LEfSe first performs statistical analysis to calculate significant differences among biological classes and then performs additional tests to evaluate whether the observed differences are consistent with expected biological behavior. LEfSe analysis was performed with an α value of 0.05 for the factorial Kruskal–Wallis test.

### 2.5. Statistical Analysis

Graphs were generated using GraphPad v9. Results are represented as mean ± standard error of the mean unless stated otherwise. Comparisons between two groups were performed using two-tailed unpaired *t*-test or Mann–Whitney test assuming unequal variance. Multiple comparisons were performed using one-way ANOVA, and *p* values <0.05 were considered significant.

## 3. Results

### 3.1. EEN Treatment Improves Intestinal Inflammation in the Rat Model of Crohn’s-like Colitis

**Figure 1:**
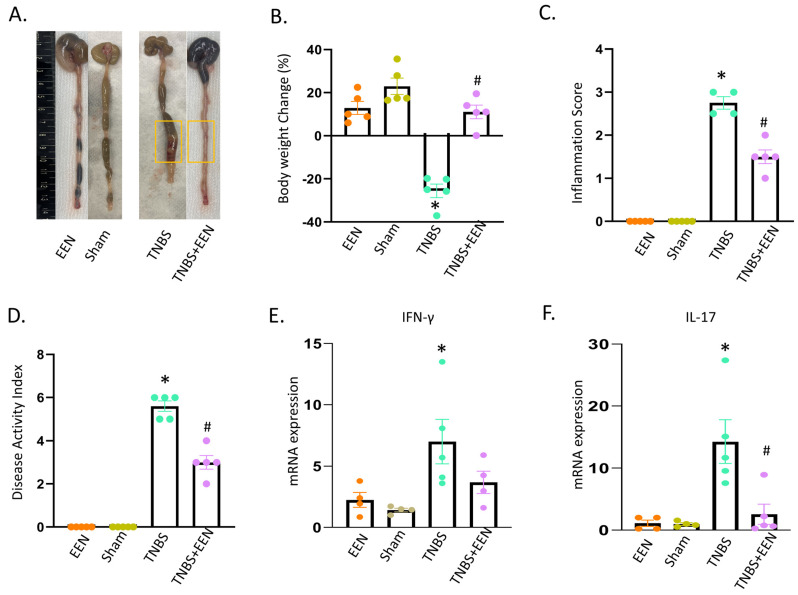
EEN reduces inflammation in the model of TNBS-induced CD-like colitis. (**A**) Representative views of rat colons in the four groups (EEN, sham, TNBS, and TNBS+EEN). The colon segment with inflammatory changes is marked with a yellow box. (**B**) Body weight changes. (**C**) Inflammation score. (**D**) Disease activity index (DAI). Gene expression levels of IFN-γ (**E**) and IL-17 (**F**) in the four groups. All rats were euthanized 7 days after induction of inflammation. EEN = control rats treated with EEN; Sham = control rats fed with regular diet; TNBS = TNBS rats fed with regular diet; TNBS + EEN = TNBS rats treated with EEN with liquid diet. N = 5. * *p* < 0.05 vs. sham control. # *p* < 0.05 vs. TNBS/regular diet.

To investigate the effect of EEN treatment with a liquid diet on intestinal inflammation, we employed a rat model of TNBS-induced CD. A previous study from our lab in the same model revealed that EEN treatment reduced inflammation; in particular, Th17 induced inflammation in CD rats [15]. Concordantly, our present study showed that EEN treatment has protective effects in TNBS rats (Figure 1). When rats were fed with regular pellet food, TNBS treatment induced stenotic inflammation in the instillation site and marked distention in the segment proximal to inflammation (yellow box). However, EEN treatment with the liquid diet reduced inflammation and released distention in the colon (Figure 1A). The body weight was significantly decreased in TNBS-induced CD rats when they were fed with regular pellet food. However, EEN administration significantly improved the body weight in TNBS rats (Figure 1B). The disease activity index and inflammatory score significantly improved in TNBS rats when fed with EEN (Figure 1C,D). To further assess the inflammatory response, we analyzed the inflammatory cytokine gene expression in rat colons of all the four groups (EEN, sham, TNBS, TNBS+EEN). It was found that the mRNA levels of inflammatory cytokines such as IFN-γ (4.71 ± 0.75) and IL-17 (20.6 ± 5.4) were significantly (*p* < 0.05) increased in TNBS-induced CD rats when fed with the regular pellet diet. However, IFN-γ (2.5 ± 0.82) and IL-17 (2.56 ± 1.6) levels were not significantly (*p* > 0.05) increased in TNBS-induced CD rats treated with EEN (Figure 1E,F). Taken together, the results revealed that EEN treatment with a liquid diet alleviated intestinal inflammation in the TNBS-induced CD-like colitis model.

### 3.2. EEN Treatment Enriches the Diversity and Preserves Structure of the Gut Microbiota

As EEN treatment might alter the gut microbiome, we sought to compare the microbiota profiles in sham and TNBS-treated rats when these rats were fed with regular pellet food or a liquid diet (EEN). To achieve this, we collected the fecal samples from the distal colon in all four groups of rats (sham, TNBS, EEN, and TNBS + EEN). Bacterial DNA was isolated from the fecal samples and 16S rRNA sequencing was performed to evaluate the effects of EEN on gut microbiota.

**Figure 2:**
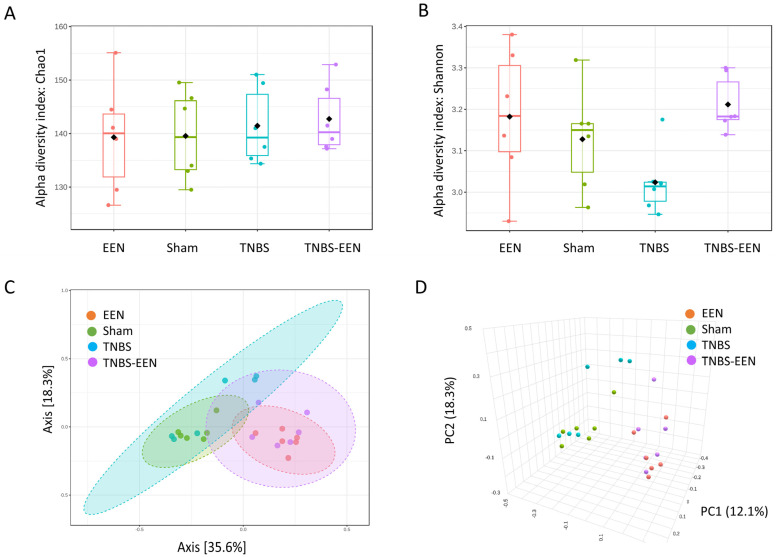
EEN treatment enriches the diversity of gut microbiota. Fecal samples were collected from the distal colon of all four groups of rats after 7 days of feeding with either regular diet or EEN treatment with a liquid diet. Alpha diversity was measured by the Chao index (**A**) and Shannon index (**B**). Beta diversity was measured by UniFrac distance-based principal coordination analysis (PcoA) and nonmetric multidimensional scaling (NMDS) (**C**,**D**). Each point represents an individual sample, and the same color indicates a specific group (n = 6 samples per group). EEN = control rats treated with EEN; Sham = control rats fed with regular diet; TNBS = TNBS rats fed with regular diet; TNBS + EEN = TNBS rats treated with EEN with liquid diet.

As shown in Figure 2A,B, EEN treatment increased microbial within-community diversity (alpha or α-diversity), represented as Chao1 and Shannon indices. Our results showed that the α-diversity was decreased in TNBS-induced CD rats as compared to sham control rats. Interestingly, EEN treatment increased the α-diversity in TNBS rats (Figure 2A,B). On the other hand, analysis of the between-community diversity (beta or β-diversity), performed using UniFrac distance-based principal coordination analysis (PCoA) and nonmetric multidimensional scaling (NMDS), revealed that OTUs from the sham control and CD rats fed with the regular diet overlapped. It is also evident that the OTUs from the sham and CD rats fed with EEN were also overlapped. The distinct separation of EEN- and regular-diet-fed rats suggests a significant difference in the gut microbiota structure between the two cohorts (Figure 2C,D). Collectively, these data indicate that EEN treatment rigorously changes fecal microbial α- and β-diversity compared with rats fed with regular solid food.

### 3.3. EEN Treatment Alters the Bacterial Composition at Phylum and Genus Levels

**Figure 3:**
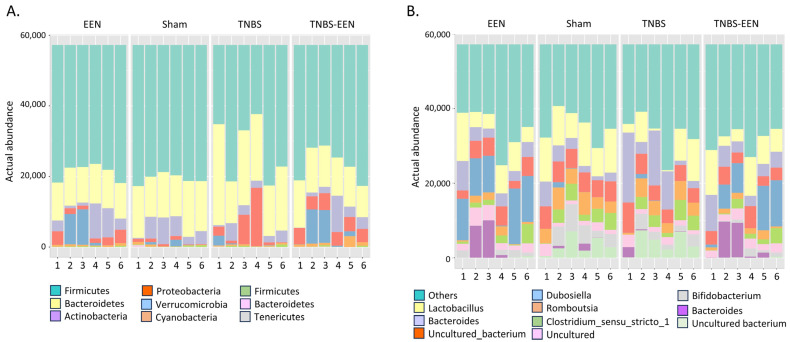
EEN treatment changes the compositions of the gut microbiota. The microbial composition at phylum level (**A**) and genus level (**B**) are shown. Fecal samples were taken 7 days after induction of inflammation. N = 6 samples per group. Shown are data of each individual animal in the groups (1–6). EEN = control rats treated with EEN; Sham = control rats fed with regular diet; TNBS = TNBS rats fed with regular diet; TNBS + EEN =TNBS rats treated with EEN with a liquid diet.

The dominant bacterial phyla in the gut of healthy individuals are Firmicutes, Bacteroidetes, Proteobacteria, and Actinobacteria. However, various studies in adult CD patients have demonstrated that the intestinal microbiota is characterized by an increased abundance of Proteobacteria and a decrease in Firmicutes and Bacteroidetes compared to healthy individuals [26]. To determine whether gut microbiota composition is altered in our rat model of CD-like colitis and, if so, whether EEN treatment improves it, we analyzed the changes in gut microbial composition at the phylum and genus levels among the four different groups (EEN, Sham, TNBS, TNBS-EEN). We found that 98–99% of the gut microbial community was dominated by Firmicutes, Bacteroidetes, Actinobacteria, and Proteobacteria in all four groups. However, the composition of these dominant phyla was different among the groups. The TNBS group (when fed with regular diet) displayed an increased level of Bacteroidetes and Proteobacteria. Firmicutes remained as the dominant phyla in the three other groups. Interestingly, EEN-fed groups (EEN and TNBS-EEN) showed increased Verrucomicrobia and Actinobacteria, as compared to regular-food-fed groups. In terms of lower taxonomic levels, EEN feeding for 7 days to TNBS rats altered the phylum structure, as shown in Figure 3A.

At the genus level as shown in Figure 3B, we observed that *Lactobacillus* genera were decreased in TNBS-induced CD rats compared to the other three groups. With EEN feeding in TNBS rats, *Lactobacillus* increased to various degrees (Figure 3B). However, EEN feeding to both sham and TNBS rats revealed an increased abundance of genera *Dubosiella* and *Akkermansia* relative to the regular-diet-fed sham control and TNBS rats. These data depict differential effects of EEN in the gut microbiome at both phylum and genus levels.

**Figure 4:**
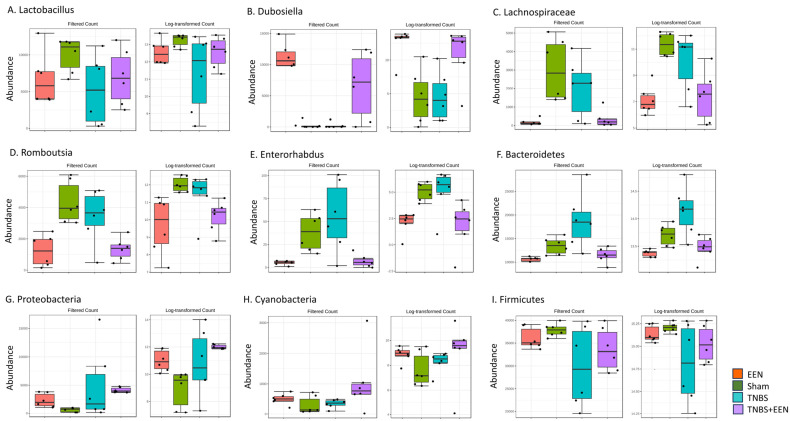
Comparison of the significantly altered genus and phylum among the four groups of rats. Samples were taken 7 days after induction of inflammation. Shown in panels a–f are the relative abundance of *Lactobacillus* (**A**), *Dubosiella* (**B**), *Lachnospiraceae* (**C**), *Rumbotsia* (**D**), *Enterorhabdus* (**E**), and *Bacteroidetes* (**F**). Relative abundance at phylum level was also analyzed for the following: Proteobacteria (**G**), Cyanobacteria (**H**), and Firmicutes (**I**). N = 6 samples per group. EEN = control rats treated with EEN; Sham = control rats fed with regular diet; TNBS = TNBS rats fed with regular diet; TNBS + EEN = TNBS rats treated with EEN with a liquid diet.

Comparison of the relative abundance at the genus levels among different groups revealed a significantly increased abundance of genera *Lactobacillus* and *Dubosiella* in EEN-fed groups compared to regular-diet-fed groups (Figure 4A,B). However, genera *Lachnospiraceae, Enterorhabdus, Romboutsia,* and *Bacteroidetes* were reduced in EEN-fed groups (Figure 4C–F). At the phylum level, Proteobacteria and Cyanobacteria showed an enriched abundance in EEN-fed groups relative to regular-diet-fed groups (Figure 4G,H). However, the composition of the dominant phylum Firmicutes is not different among all the four groups (Figure 4I). These results depict that EEN feeding has enriched beneficial bacterial species such as *Lactobacillus* and *Dubosiella* and reduced pathogenic bacteria like *Enterorhabdus*.

**Figure 5:**
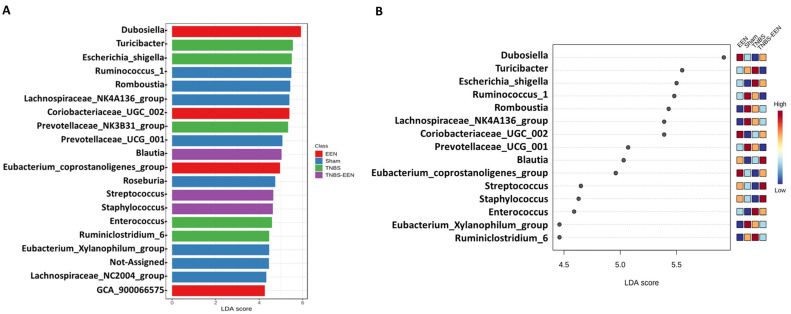
LEfSe analysis of the abundant genus among different groups. Plots (**A**,**B**) show LDA scores of the taxa with significant effects. Differences were shown by the color of the most abundant class. N = 6 samples per group. EEN = control rats treated with EEN for 7 days; Sham = control rats fed with regular diet; TNBS = TNBS rats fed with regular diet; TNBS + EEN = TNBS rats treated with EEN with a liquid diet.

Finally, we performed linear discriminant analysis effect size (LEfSe) to identify the differentially abundant taxa with EEN feeding at phylum to genus levels among the four groups. The results, depicted in Figure 5A,B, show that *Dubosiella*, *Coriobacteriae*, *Blautia*, *Streptococcus*, and *Staphylococcus* are the important components of the gut microbiome in rats fed with EEN. Notably, IBD-associated *Escherichia Shigella* is an important component in the gut microbiota of TNBS rats fed with a regular pellet diet. Taken together, the results of the present study reveal an EEN-specific microbial signature in both sham and TNBS-induced CD rats.

## 4. Discussion

In the present study, we demonstrated that EEN treatment improved intestinal inflammation along with the beneficial modulation of the gut microbiome in a rodent model of Crohn’s-like colitis induced by an intracolonic instillation of TNBS. Specifically, EEN with a liquid diet led to improved body weight changes, disease activity index, inflammation score, and reduced pro-inflammatory gene expression along with microbial correction in TNBS rats. Microbiome analysis revealed that TNBS rats had microbial dysbiosis, when they were fed with regular pellet food. Microbial dysbiosis and immune dysregulation leading to impaired intestinal homeostasis is a prominent characteristic of CD. Gut microbial components and their derived metabolites contribute widely to gut homeostasis. The association between microbiome dysbiosis and CD has been demonstrated by various studies comparing the differences between healthy and CD patients [3,26]. It is found that the dominant phyla such as Firmicutes and Bacteroidetes are reduced in CD patients, whereas Proteobacteria abundance is increased [26]. Bacterial strains such *Escherichia coli* and *Shigella* are prominent in the microbiome of CD patients [3]. The 16s rRNA sequencing in our study revealed similar results with increased Proteobacteria and *Enterorhabdus* abundance in the fecal content of TNBS rats as compared to sham control rats.

There have been several scientific attempts to correct the microbial dysbiosis in IBD by targeting whole or individual pathogenic bacterial species involved with disease progression [27]. These strategies include supplementing with probiotics, prebiotics, and fecal microbiota transplantation (FMT) [28] to achieve CD remission. Among these various strategies, dietary treatments such as EEN have shown promising outcomes in the management of CD [29]. EEN is an established dietary treatment consisting of complete nutritional components. It is a liquid diet treatment avoiding any solid food for 6–8 weeks. The use of EEN for the treatment of pediatric CD was first reported in the 1980s [30]. EEN treatment has been reported to show 80% of remission in pediatric CD patients [5]. Although EEN is an effective dietary treatment, the mechanism of action is not yet clear. In the present study, to investigate the mechanism of action of EEN, we fed control and TNBS rats with or without EEN. We found that TNBS rats fed with a regular diet showed characteristics of CD. The EEN treatment of TNBS rats not only reduced inflammation but also corrected the gut dysbiosis. Control rats with EEN treatment did not show any significant difference in inflammatory gene expression, indicating that EEN feeding to control rats does not change the inflammatory microenvironment. Interestingly, fecal microbiota analysis of the TNBS-EEN group showed increased levels of α-diversity as compared to the TNBS-regular diet group. This result is in contradiction with few previous reports which demonstrated that EEN treatment reduced microbial α-diversity in CD patients. Leach et al. found that EEN treatment reduced the diversity and decreased the relative abundance of the *Bacteroides* genus and *Clostridium Coccoides* species in pediatric CD patients [31]. Few other studies corroborated with the observation that EEN feeding led to a reduction in bacterial diversity, creating a community structure even more dissimilar than that of controls [32,33]. Gerasimidis et al. studied the EEN effect on microbial metabolite butyrate, a beneficial short-chain fatty acid, and found that butyrate was decreased after EEN treatment [33]. Contrary to our results, those studies showed harmful effects of EEN on the gut microbiome and could not explain the therapeutic benefits of EEN in CD.

Our study suggests that the therapeutic efficacy of EEN for CD may be partly due to its beneficial effects on the gut microbiome, as EEN increases the α-diversity of the microbiome community in TNBS rats. It is difficult to explain exactly what accounts for the differences between our and the abovementioned studies. However, we carried out this study in a well-controlled and standardized animal model of Crohn’s-like colitis, where 8–9-week-old rats were given an intracolonic instillation of TNBS at 65 mg/kg in 40% of ethanol. All other abovementioned results were from patients with a wide range of ages. The degrees of inflammation and extents of the disease process might also be very different among patients. It would be difficult to ensure compliance with the EEN protocol among all patients. The nutrient compositions and liquid diets used for EEN treatment may be very different among patients and between the studies. In our study in the animal model, EEN with a liquid diet was always applied strictly after the induction of inflammation, and rats were housed in wire-bottomed cages to prevent them from eating feces or anything at the bottom of the cages. As previous studies reported that EEN decreased the microbial diversity, the role of microbiome remodeling in EEN-mediated CD remission has been questioned. Nevertheless, our study has revealed a distinct microbial signature to the EEN-fed group compared to regular diet groups. PCoA evidently revealed the separation of the gut microbiome in the EEN- and regular-diet-treated groups. The differences in relative abundance levels of various phyla and genera in these groups are illustrated using bar plots and heat maps. The beneficial bacteria such as *Lactobacillus* and *Dubosiella* were significantly increased in the EEN-fed group. Interestingly, opportunistic pathogens such as *Escherichia–shigella* and *Enterorhabdus* were significantly reduced in CD rats fed with EEN. These structural changes are in corroboration with several previous studies in humans, which showed beneficial effects of EEN on the gut microbiome [34,35,36]. Recently, Hart et al. observed the effects of EEN treatment on microbiota and inflammation for 8 weeks in pediatric patients with active CD [36]. Patients showed a significant increase in Shannon diversity over the 8 weeks of treatment. Importantly, a higher increase in diversity indices detected as early as week 2 was associated with remission, while low or no increase in diversity by week 2 was found in patients who continued to have active disease by week 8. Collectively, the effect of EEN on gut microbiome diversity and compositions may partly account for its beneficial action on inflammation in CD.

Currently, we do not know what accounts for the effects of EEN on the microbiota diversity and composition observed in our study. On the one hand, components of the liquid diet may have contributed to the effect. We used a clear liquid diet from Ensure in our study. The studies which observed a reduced diversity of microbiota used different liquid diets [31,32]. However, the nutritional compositions in these liquid diets are like the diet we used in our study. To better understand if specific nutrient components contribute to microbial changes, further studies are warranted to delete specific components in the diet or to test individual components for the effects on gut microbiota. On the other hand, the liquid nature of the EEN diet and the release of obstruction by the liquid diet may also play a role in the microbiota changes observed in our study. Our colitis model represents a stenotic inflammation with corresponding lumen distention. The microbial dysbiosis (i.e., increased proteobacteria, decreased firmicutes, and decreased alpha diversity) in the distended lumen of TNBS colitis rats is very similar to that observed in a mechanical obstruction model [19]. However, EEN treatment with a liquid diet reduced lumen distention [15] and corrected the microbial dysbiosis. Thus, the observed microbiota changes by EEN treatment may be partly due to the release of mechanical distention in the colon.

Although our study has detected important changes at the genus level with EEN treatment, we do not know exactly what microbial changes account for the reduced inflammation in the CD model. However, an increase in abundance of genera such as *Lactobacillus* and *Dubosiella* may play a role. *Lactobacillus* is one of the prototypical bacteria utilized in probiotics for IBD and other types of gut inflammation [37]. It was found that multiple strains of lactobacillus alleviated intestinal inflammation and improved barrier function and the mucus barrier in the damaged intestine in IBD [37]. The *Dubosiella* genus is shown to increase the levels of superoxide dismutase, an antioxidant enzyme. Antioxidant compounds like resveratrol [38] and dihydroquercetin [39] were found to modulate the microbiome by increasing the levels of *Dubosiella*, *Lactobacillus*, and *Bifidobacterium*. Wan et al. found that an increased abundance of *Dubosiella* is negatively correlated with pro-inflammatory cytokine expression in a colitis model [39]. Further studies on *Lactobacillus* and *Dubosiella* may help develop therapeutic strategies for CD.

While the positive effect of EEN on the gut microbiome may contribute to the therapeutic efficacy of EEN, few other mechanisms have been proposed to underlie the benefits of EEN in CD [7,8,9,10]. Earlier studies suggested that EEN is beneficial in CD by regulating the intestinal inflammatory responses by improving barrier function in the gut [11]. However, the mechanism is not known. CD is characterized with transmural inflammation and stenosis, which lead to lumen distention and mechanical stress in the affected tissues. In a recent study, we reported a novel mechanism of action of EEN in CD-like colitis, as EEN treatment with a liquid diet dramatically eliminated mechanical stress in the model. We presented evidence that the elimination of mechanical stress in the gut contributes to the attenuation of inflammation and immune response. Our studies in vivo and in vitro showed that mechanical stress induced pro-inflammatory cytokine IL-6 in the colon, and IL-6 promoted differentiation of the Th17 immune response. Importantly, EEN treatment eliminated mechanical stress and attenuated IL-6 up-regulation and the Th17 immune response in rats with CD-like colitis [15]. Taken together, EEN treatment reduces inflammation possibly through multiple mechanisms by improving barrier function, relieving mechanical stress, and remodeling the gut microbiome.

## 5. Conclusions

In summary, the present study demonstrates that EEN feeding to control and TNBS rats increased the microbiome diversity and improved the microbial composition in the colon. These changes may contribute to the beneficial effect of EEN in TNBS-induced Crohn’s-like colitis. This work, together with previous studies, suggests that the beneficial effects of EEN on CD are mediated through various mechanisms such as eliminating mechanical stress, improving barrier function, and remodeling gut microbiota.

## Data Availability

Data are contained within the article.
